# Broad-Spectrum Theranostics and Biomedical Application of Functionalized Nanomaterials

**DOI:** 10.3390/polym14061221

**Published:** 2022-03-17

**Authors:** Meshal Alshamrani

**Affiliations:** Department of Pharmaceutics, College of Pharmacy, Jazan University, P.O. Box 114, Jazan 45142, Saudi Arabia; malshamrani@jazanu.edu.sa

**Keywords:** theranostics, bioconjugation, drug delivery systems, biomedical imaging, tissue engineering

## Abstract

Nanotechnology is an important branch of science in therapies known as “nanomedicine” and is the junction of various fields such as material science, chemistry, biology, physics, and optics. Nanomaterials are in the range between 1 and 100 nm in size and provide a large surface area to volume ratio; thus, they can be used for various diseases, including cardiovascular diseases, cancer, bacterial infections, and diabetes. Nanoparticles play a crucial role in therapy as they can enhance the accumulation and release of pharmacological agents, improve targeted delivery and ultimately decrease the intensity of drug side effects. In this review, we discussthe types of nanomaterials that have various biomedical applications. Biomolecules that are often conjugated with nanoparticles are proteins, peptides, DNA, and lipids, which can enhance biocompatibility, stability, and solubility. In this review, we focus on bioconjugation and nanoparticles and also discuss different types of nanoparticles including micelles, liposomes, carbon nanotubes, nanospheres, dendrimers, quantum dots, and metallic nanoparticles and their crucial role in various diseases and clinical applications. Additionally, we review the use of nanomaterials for bio-imaging, drug delivery, biosensing tissue engineering, medical devices, and immunoassays. Understandingthe characteristics and properties of nanoparticles and their interactions with the biological system can help us to develop novel strategies for the treatment, prevention, and diagnosis of many diseases including cancer, pulmonary diseases, etc. In this present review, the importance of various kinds of nanoparticles and their biomedical applications are discussed in much detail.

## 1. Introduction

Nanotechnology is an important branch of science in therapies and is known as “nanomedicine”; it is the junction of various fields such as material science, chemistry, biology, physics, optics, etc. [1]. Recently, many new investigations on nanotechnology have been conducted to enhance the efficacies of therapies, drug delivery, imaging, and sensors due to nanoscale sizes and the shapes of nanomaterials possessing unique properties such as optical, magnetic, mechanical, and chemical relativities. Nanotechnology helps society with early detection, the prevention of diseases, effective drug delivery and the development of effective and low-cost home-based healthcare. Various biomolecules such as small molecules, peptides and proteins have also been used for imaging and therapeutic agents. Exosomes, liposomes, micelles, nanoemulsions, nanoparticles and dendrimers have been extensively used for drug delivery and imaging [2].

**Figure 1:**
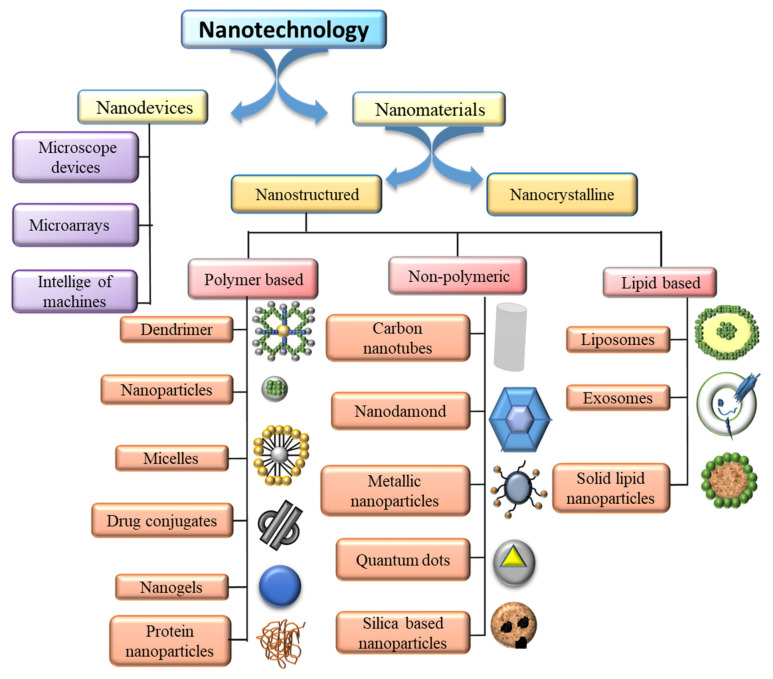
Classification of various kinds nanomaterials used for therapeutic applications.

Nanomaterials range between 1 and 100 nm in size and provide a large surface area to volume ratio. They can be used for various diseases, including cardiovascular diseases, cancer, bacterial infections, and diabetes [3]. Nanoparticles, with their unique magnetic, electrical, and optical properties, can be used for various imaging modalities and are also often conjugated with ligands or various functional materials for the development of multimodal imaging, immunoassay tissue engineering and therapies [4]. Based on their structure, nanomaterials can be categorized into nanostructured and nanocrystallinematerials. Nanostructured materials can be further classified, based on the type of material used, as polymer-based, non-polymer-based and lipid-based. Polymer-based nanoparticles include dendrimers, micelles, nanogels, etc., whereas non-polymer-based nanoparticles include carbon nanotubes and metallic nanoparticles. On the other hand, lipid-based nanoparticles involvesolid lipid nanoparticles, liposomes, exosomes, etc. (Figure 1). The crystalline form of nanoparticles is also used intherapeutic applications [5]. Nanomaterials can either be added to the preexisting product for improving the performance of the product or used to create the new devices/products [6]. In this review, we will be discussing the types of nanomaterials that have various applications. Nanoparticles play a crucial role intherapies as they can enhance the accumulation and release of pharmacological agents, improve targeted delivery, and ultimately decrease the intensity of the side effects of the drug. Nanoparticles, through their diagnostic and therapeutic potentials, could be effectively used for monitoring the distribution and accumulation of drugs and could also beused for the quantification and visualization of the drugs’ release, thereby improving their therapeutic potential [7]. The FDA has approved nanoparticle-based products such as liposome, micellar, and protein nanoparticlesin combination with drugs or with any biological molecule [8].

The bioconjugation of nanoparticles is the interaction of biological molecules with nanoparticles. This enhances the assembly of the nanoparticles and modulates the potential of nanoparticles with specific recognition or detection efficacy. The bioconjugation of nanoparticleshas been extensively used in the fields of biodetection, nanomedicine, nanosensors [9]. Biomolecules that are often conjugated with nanoparticles are proteins, peptides, DNA, and lipids, which can enhance biocompatibility, stability, solubility, etc. Bioconjugation with nanoparticles involves four important processes including covalent binding, non-covalent binding (affinity-mediated), electrostatic binding and chemisorption. As nanoparticles act as the foundation of nanotechnology, their application in the field of medicine provides a new strategy for the treatment of various diseases. In this review, we have focused on bioconjugation and nanoparticles, and discussedtheir crucial role in clinical applications such as medical devices, bio-imaging, drug delivery, biosensors, tissue engineering, etc.

## 2. Types of Nanoparticles

Nanoparticles, with their nanoscale rangeeffectively interacting with the biological system, are used to develop a sensor system to detect and diagnose diseases and help with revealing the body conditions of patients. Cell membrane-based nanoparticles can be in the body without causing damage to the biological system and several nanoparticles are developed to carryhigh doses of drugs or target certain cells or tissues to enhance biocompatibility and biodegradability at a reasonable cost, with renewable resources, etc. In this section, we will be discussing nanoparticles and their uses.

### 2.1. Micelles

**Figure 2:**
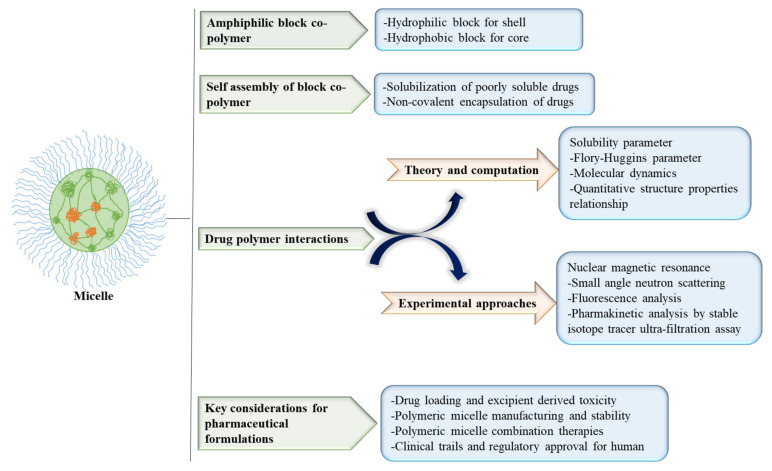
Use of Micelle nanoparticles for efficient drug delivery system and their advantages.

Micelles are self-assembled amphiphilic surfactant molecules with a diameter ranging from 10 to 100 nm. They consist of a hydrophilic shell and a hydrophobic core under aqueous conditions, and could be used to incorporate hydrophobic therapeutic agents. The hydrophilic shell prevents drug loss and avoids the opsonization process via a complement system which leads to the rapid clearance of drugs from systemic circulation [10]. Micelles act as carriers by enhancing the solubility of hydrophobic therapeutic agents, thereby improving bioavailability [11]. Polymeric micelles play a crucial role in reducing the toxicity of therapeutic compounds such as hydrophobic drugs, small molecule drugs, proteins, DNA, siRNA, peptides and photosensitizers [12]. The use of bioconjugated micellenanoparticles is shown in Figure 2. The self-assembly depends on polymer chain concentration, drug properties, and the composition of the copolymer backbone. Based on the molecular weight, micelles can have cylindrical and star-shaped structures [13]. It has been reported that polymeric micelles overcomeocular barriers and prevent adverse effects of drug formulations [14]. Therapeutic agents can be encapsulated into polymeric micelles by various processes including direct dissolution, dialysis, oil-in-water emulsion, solvent and co-solvent evaporations, and freeze-drying methods [15]. Some of the in vitro and in vivo data on drug encapsulation in micelles and their roles have been provided in this section.

In a study, an MMP-2/9-sensitive micelle peptide-conjugated polymerwas developed to improve tumor targeting, accumulation and enhanced loaded drug release at tumor locations, thereby stimulating the antitumor effect both in vitro (MCF-7 and HT1080) and in vivo(tumor-bearing nude mice modelHT1080) [16]. The resveratrol derivative is 3,5,4′-trimethoxy-trans-stilbene (BTM) and it is loaded in the PEG–PE micelles acting against colon cancer cells (CT26 cells) reporting anti-cancer efficacy and in tumor-bearing mice with an improved half-life and increased bioavailability [17]. Paclitaxel was effectively delivered to the tumor site with the help of the folic acid-hyaluronic acid-SS-vitamin E succinate polymer which enhanced selective targeted toxicity to the cancer cells without damaging healthy cells by analyzing cytotoxicity, apoptosis and cellular uptake studies in MCF-7 and NIH3T3 cells [18]. Studying the retention effects and skin penetration of thiolatedpluronic f127 polymeric micelles loaded with berberine using in vitro transdermic and fluorescence microscopy analysis showed that preparations enhanced the therapeutic benefit by decreasing the entry of drugsinto the systemic circulation, thereby preventing side effects and effectively treatinglocal skin diseases [19]. Curcumin, with effective anticancer potential, possess poor solubility and bioavailability and it was reported to be improved by using PEG–PLA copolymer micelles that act as an effective nanocarrier of curcumin, which could be used for treating non-small lung cancer cells [20].

The application of low-frequency ultrasound (20–100 kHz) enhances the penetration of drugs and acts as an energy source for the sonodynamic therapy of skin cancers. Due to poor water solubility and decreased penetration, ZnPc molecules are encapsulated in DSPE–PEG micelles and subjected to skin cancer treatment. Data showed that the low-frequency ultrasound irradiation of ZnPc-loaded micelles generates reactive oxygen species and stimulates lipoperoxidation in the skin that would eventually lead to tumor cell death [21]. Similarly, Liu et al. [22] reported that the encapsulation of *meta*-tetra(hydroxyphenyl)chlorine, a photosensitizer, in P23 micelles effectively increased blood circulation kinetics and ultimately enhanced selective photocytotoxicity on A431 cells. As with other studies reporting that multidrug resistance is effectively reversed by combining gene therapy and chemotherapy, Liang et al. [23] reported that the construction of AS1411 aptamer-functionalized micelles by encapsulating doxorubicin and miR-519c enhanced active targeting and suppressedthe multidrug resistance in the hepatocellular carcinoma cell line (HepG2).Apart from cancer, micelles can also be used in the treatment of other diseases such as cardiac toxicity, rheumatoid arthritis, etc. After myocardial infarction, the recruitment of monocytes to the infract through the CCR2 chemokine receptorprolongs inflammation and worsens clinical outcomes. To avoid this, a study was conducted to develop PEG–DSPE micelles with a small molecule of the antagonist CCR2 that would effectively prevent the recruitment of monocytes to the infarcted myocardium. According to the study, in a mouse model of myocardial infraction using C57BL/6 mice, PEG–DSPE micelles witha small molecule of the antagonist CCR2 effectively reduced the number of inflammatory cells and decreased the infarct size when compared to the PBS-treated controls, ultimately suggesting that PEG–DSPE micelles effectively improved cardiac function and could be used in a cardiac therapeutic platform [24]. In one study, micelles of polysialic acid (PSA) were used for dexamethasone (Dex)-targeted delivery and studied for their anti-inflammatory effect. The data showed that in arthritic mice, PSA micelles effectively improved the Dex uptake by cells and decreased the levels of interleukin-6 (IL-6) and tumor necrosis factor-α (TNF-α), thereby suppressing inflammation [25]. Apart from therapy, some micelles have been used as theranostics. In one study, a novel mPEG-SS-Poly (AEMA-*co*-TBIS) was loaded with doxorubicin (DOX). The data showed that micelles provided the potential for two-photon cell imaging and deep tissue imaging and additionally provided the targeted delivery of DOX at the tumor site, thereby enhancing the antitumor effect [26].

### 2.2. Liposomes

**Figure 3:**
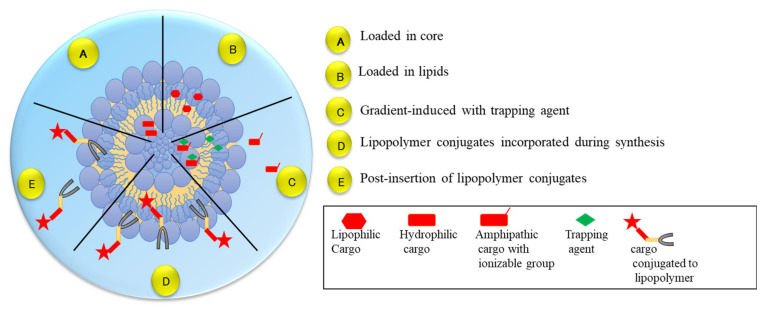
Illustartion of bioconjugated lipid-based nanoparticles for drug delivery.

Liposomes are spherical vesicles with sizes ranging from 100 to 500 nm to several microns and are formed from the self-assembly of phospholipids that consistof a hydrophilic head and hydrophobic tail. Under aqueous conditions, the hydrophobic tail undergoes self-assembly and leads to the formation of a spherical structure consisting of an aqueous core surrounded by a liposomal membrane layer [27]. Liposomes act as both drug carriers and delivery agents, and the first FDA-approved nanomedicine was liposomal doxorubicin (Doxil^®^) which was used for breast cancer treatment in 1995 [28]. Liposomes are widely used to deliver hydrophilic therapeutic drugs and their surface can be modified with polymers, antibodies, proteins, etc. (Figure 3). Therapeutic agents or small molecules can be incorporated into the hydrophilic core or can be encapsulated within the liposomal membrane layer or a combination of both can be performed to enhance targeted delivery and to improve tissue and cellular imaging [29]. Because of their nanoscale potential, they exhibit enhanced solubility, bioavailability, and diffusion with less immunogenic and biodegradable properties [30]. Liposomes are produced by various methods including reverse-phase evaporation, thin-film hydration, freeze–thaw methods, polyol dilution, ethanol injection, detergent removal and high-pressure homogenization [30,31]. Liposomes are classified based on their preparation (i.e., the number of bilayers formed in liposomes) and on their size. In this section we will be discussing the role of liposomes in various disease conditions.

In cancer-bearing nude mice and triple-negative breast cancer cell studies, microRNA was designed to slug gene silencing and was simultaneously encapsulated into peptide-modified functional liposomes. Their effect was compared with functional vinorelbine liposomes or with combined therapy. The study showed that functional liposomes via inhibiting the TGF-β1/Smad pathway improved the anticancer effect. Comparatively, combined therapy resulted in the almost complete inhibition of tumor growth and enhanced the drug half-life in blood with the increased accumulation of the drug in the breast cancer tissues [32]. In another study, it was reported that multifunctional targeting vinorelbine plus tetrandrine liposomes are effectively involved in treating brain glioma by increasing the transport of drugs across the blood–brain barrier, increasing cellular/cancer tissue uptake, inhibiting the growth of glioma cells and stem cells, increasing apoptosis and enhancing specificity to the tumor in both in vitro and in vivo models [33]. Alaarg et al. [34] reported that the ω3 fatty acid–docosahexaenoic acid–liposome formulation effectively inhibited the release of reactive nitrogen species and reactive oxygen species from neutrophils and macrophage cells. It also significantly inhibited the synthesis of the proinflammatory cytokines including TNFα and MCP1 and it suppressedthe cell proliferation of head and neck and breast cancer cells, ultimately suggesting that liposomes could be effective agents in the treatment of inflammatory disorders and cancer [34]. In one study, the photodynamic therapeutic drug 2-(1-Hexyloxyethyl)-2-devinyl pyropheophorbide-a (HPPH) activated by red lightwas encapsulated in pocket lysosomes along with calcein as a marker for drug release and tested against breast cancer cells and xenograft mice. The study reported that there was a significant decrease in the cell growth and tumor volume, with enhanced the tumor necrosis of breast cancer cells [35].

A membrane dispersion-ultrasonic method was used to develop the liposome with the red cell membrane and was modified with the peptides TAT and PCM. This method tested for the uptake efficiency and cellular toxicity of myocardial cells and in heart tissue. Liposomes were reported to exert the best delivery capability with a prolonged mean residence time and enhanced accumulation in the heart. This suggests that liposomes could act as effective myocardial drug delivery systems and could help in various heart diseases including ischemic cardiac disease [36]. Krill oil, an omega-3 rich marine-derived oil, is effectively incorporated into liposomes (KO liposomes) and showed a high capacity to budesonide (BDS) entrapment in an intestinal bowel disease (IBD) drug. In the same study, it was reported that KO liposomes effectively inhibited the lipopolysaccharide-induced synthesis of cytokines’pro-inflammation in macrophage cultures and also restored the complete inflammation of the impaired membrane barrier function in an intestinal barrier model. Additionally, in the colitis mouse model, the oral administration of BDS-entrapped KO liposomes inhibited inflammation by inhibiting the synthesis oftumor necrosis factor-α, interleukin-6, and endotoxin (by 96.8%), and acts as an essential platform for the nanovehicle for IBD therapy [37]. Kuo et al. [38] reported that liposomes developed with cardiolipids and wheat germ agglutinin effectively passage curcumin and the nerve growth factor across the blood–brain barrier (BBB) for the protection of SK-N-MC cells against β-amyloid fibril induced by apoptosis and could act aseffective colloidal delivery carriers to cross the BBB and treat Alzheimer’s disease. Vanić et al. [39] developed Azithromycin-liposomes to test against planktonic and *Escherichia coli* strains supporting biofilm formation and *Chlamydia trachomatis* intracellular infection and suggested that AZI-liposomes and cervical cells are biocompatible. Moreover, they also suggested that drug localization is improvedwithin the vaginal tissue while enhancing antibacterial activity by preventing *E. coli* 700928, inhibiting the formation of *E. coli* 8739 biofilmsand eradicating *E. coli* biofilms. As an autoimmune disorder, type 1 diabetes leads to β-cell destruction which produces insulin. Rodriguez-Fernandez et al. [40] reported that the development of liposomes with phosphatidylserine and β-cell autoantigensmimics apoptotic β-cells which leads to the inhibition of autoimmunity to β-cells, thereby effectively preventing experimental type 1 diabetes through the generation of tolerogenic dendritic cells.

### 2.3. Dendrimers

Dendrimers (1–5 nm in size) are macromolecules with globular structures, consist ofbranched repeating layers starting from a core in the center, contain terminal exterior functional groups, and finally resemble a tree-like structure. Compared to liposomes, dendrimers can bound covalently with drugs, are stable and can improve the solubility of drugs. The exterior functional groups reported are cationic, neutral or anionic terminals, which help in the modification of the entire structure, and/or the physical and chemical properties of dendrimers [4,41]. Dendrimers are used for therapeutic agent delivery; for RNA, DNA, and protein delivery; and also for imaging. Dendrimers, by covalent bonds, ionic interaction and the adsorption process, can effectively carry therapeutic or biomolecules with low cytotoxicity enhancement and bio-permeability of the molecule at a high level [5]. Dendrimers can also be linked with other polymers including PEG together with other biomolecules such as aptamers, antibodies, folic acid and carbohydrates, which can be used for various therapeutic applications [42]. Various in vivo and in vitro studies have been conducted to understand the significance of dendrimers and are discussed in this section.

Nanoparticles, especially dendrimers, are reported to effectively cross the blood–brain barrier and efficiently deliver medication into the central nervous system. Novel dendrimers (G4-90/10 and G1-90/10) and amine dendrimers (G1-NH2 and G4-NH2) were tested for the uptake of dendrimers in primary cortical cells. G4-90/10 significantly crosses the BBB and enters neurons and glial cells upon injection through the carotid artery which ultimately suggests that poly-amidoamine (PAMAM) dendrimers can be used for biomolecule delivery for the treatment of neurological diseases or disorders [43]. Sulfonated naphthyl (G2-NF16) and sulfated anionic carbosilane dendrimer (G3-S16)-ended groups with different antiretrovirals were tested against HIV-1 infection and it was found thatG2-NF16 and G3-S16 with compounds of zidovudine, efavirenz, and tenofovir enhanced the antiviral potency [44]. Along with PEG2K, PEGylatedG4dendrimer with a DNA ratio of 20:1 evidentlyreduced the B16F10-Luc cells’ cytotoxicity and effectively acts in cancer therapy as a gene delivery systemwith a low level of cytotoxicity and a high level of transfection efficacy [45]. Trastuzumab (TZ)-grafted dendrimers loaded with docetaxel (DTX) were tested against HER2-positive breast cancer cells and the data showed that dendrimers were more selective with the enhanced site-specific delivery of DTX, had less systemic toxicity, increased the anti-proliferation potential, increased cellular internalization, enhanced apoptosis inMDA-MB-231 and MDA-MB-453 human breast cancer cells, and could efficiently be used in breast cancer patients [46]. Polyfect (PF), a G6 PAMAM dendrimer, althoughnot activated, exerts time- and dose-dependent ERK1/2,EGFR inhibition and the phosphorylation of p38 MAPK in HEK-293 cells and can also effectively modulate in vivo EGFR signal transduction pathways, ultimately suggesting that it could act as a novel class of EGFR modulators [47].

Carbosilane dendrimers are widely used as carriers of drugs and genes due to their monodispersity, stability, and multivalence characteristics. However, the synthesis of ruthenium carbolsilane dendrimers showed increased anticancer efficiency by decreasing the viability of leukemia cells with less toxicity toless malignant cells (PBMC) and induced cancer cell death via stimulating the apoptotic pathway, suggesting that in comparison with carbosilane dendrimers, ruthenium carbosilane dendrimers could enhance the survival of cancer patients through their anti-cancer potential [48]. The dendrimerPAMAM, coupled to either 6′-sialyllactose (6SL) or 3′-sialyllactose (3SL), effectively exerted an antiviral effect by inhibiting the influenza virus, suggesting that it could be effectively used as an influenza virus inhibitorin the near future [49]. Kambhampati et al. [50] reported that ischemia/reperfusion (I/R) injured eyes retained cyanine dye (Cy5)-conjugated dendrimers (D-Cy5) for 21 days via intravitreal and intravenous administration and also showed increased retention of C-Cy5 with enhanced retinal biodistribution; they showed it could be developed as a drug formulationthatcould be used for the patients of diabetic retinopathy, retinal degenerations, etc. [51]. Manganese (MN) acts as a clinically translatable alternative to gadolinium-based agents when it exerts toxicity. In a study, Mn G8 dendrimers were synthesized and targeted to oxidative-specific epitopes and it was observed that Mn was accumulated within atherosclerotic lesions with increased Mn deposition at the arterial wall, and it was suggested that Mn G8 dendrimers could be used for the in vivo detection and imaging of atherosclerotic lesions [52]. In a study, G3.0 PAMAM dendrimers layered on the surface of dentin infiltrated into dentinal tubules and stimulated the formation of hydroxyapatite and induced the remineralization of dentin and ultimately ledto effective dentinal tubule occlusion. This paves the path towards the development of a new treatment method for dentin hypersensitivity therapy [53].

### 2.4. Carbon Nanotubes

Carbon nanotubes (1 nm in diameter and 1–100 nm in length) are carbon-based well-ordered cylindrical molecules that consist of a single-layer of carbon atoms (grapheme sheet) rolled into a cylinder. The configuration of carbon nanotubes includes multi-walled or single-walled configurations, or nanotubes interlinked concentrically that are composed ofC60 fullerenes [5]. Due to their high external surface area, mechanical strength, thermal and electrical conductivities, and exceptional thermal and chemical stability, carbon nanotubes possess high loading capacities as non-polymeric drug carriers, as imaging contrast agents and biological sensors [1,41]. Fullerenes, due to their increased number of conjugated double bonds, change in the graphite cylinder arrangementand are most often used in the delivery of therapeutic agents such as anti-cancer agents, and antibiotic and anti-viral agents [54,55]. Among carbon nanotubes, single-walled carbon nanotubes (SWCNTs) hold a specific characteristic such as having a diameterranging from 0.7 to 3 nm and thin flexible shapes, whereas multi-walled carbon nanotubes consist of several concentric SWCNT layers with diameters ranging from 1.5 nm to 220 nm [56]. Functionalization allows the carbon nanotubes to conjugate a variety of biomolecules such as carbohydrates, proteins, peptides, and therapeutic and diagnostic devices to generate economic, vastly functional therapeutic and diagnostic strategies which are also accurate [57]. Carbon nanotubes with hollow tube-like structures holdmolecules via capillary action and adsorption and have a crucial role in drug delivery applications [58]. In this section, we further discuss the significance of carbon nanotubes in various diseases under in vitro and in vivo conditions.

In a study, single-walled carbon nanotubes were prepared as thin bundles with small linear structures (CNT-1) and bounded bundles with lengthy linear structures (CNT-2), tested for their size effects on pulmonary toxicity. CNT-1 delayed the inflammation of the lung with minimal recovery, whereas CNT-2stimulated alveolar macrophages’ cellular responses and led to the immediate inflammation of the lungpost-inhalation. Comparatively, the CNT-2 treatment inhibited cell growth, stimulated reactive oxygen species production, and enhanced MIP-1α expression, ultimately suggesting that the size of the bundles of SWCNTs are linked with pulmonary toxicity [59]. The development of carbon nanotubes by loading dexamethasone with atrial natriuretic peptide antibodies, fluorescein isothiocyanate and PEGylated multiwalled carbon nanotubesis involved in the reduction of inflammation by targeting and delivering drugs to a stroke rat model at the ischemic site, suggesting that this nanoparticle could help in the successful treatment of ischemic stroke [60]. In a study, it was reported that MWCNTs together with temozolomide, effectively induced cell lysis in RG2 glioma, suggesting thatalong with the standard treatment and adjuvant therapy, this treatment helps rats with malignant glioma implants [61].

Komane et al. [62] reported that vertically aligned multiwalled carbon nanotubes (VA-MWCNTs) were functionalized and PEGylated before dexamethasone loading and were reported to enhance the effective treatment of ischemic stroke by increasing the release of dexamethasone by 55%, 65% and 95% in pHs of 7.4, 6.5 and 5.5, respectively, at the site of injury. Carbon nanotubes (CNTs) are also used for nucleic acid delivery systems in gene therapy. Huzil et al. [63] studied the effect of metallic versus semiconducting single-wall CNTs on the rate of transfection and siRNAcellular distribution in keratinocytes of murine PAM212. The data on the interaction of cellsand ultrastructural studies showed that CNTs’ metallic single wall was involved in the delivery of siRNA both into the nucleus and cytoplasm of keratinocytes, whereas semiconducting CNTs showed delivery in only the cytoplasm, suggesting that CNTs can be used in the development of nonviral gene delivery systems in the near future. In spite of their beneficial role in various diseases, a study reported that the repeated and long-term administration of carboxylated SWNTsintravenously results in the persistent accumulation in the lungs, can cause the embolization of lung capillaries, the formation of granuloma, and can induce the NF–kappaB pathway that leads to pulmonary fibrosis. Additionally, pro-fibrotic growth factors (transforming growth factor-beta 1), pro-inflammatory cytokines (tumor necrosis factor-alpha and interleukin-1 beta), fibrotic markers(type-III collagens, matrix metalloproteinase-2, type-I collagen and metalloproteinase-2), and a tissue inhibitor were reported as having high post-exposure expressions after the intravenous administration of c-SWNTs, suggesting that cumulative and chronic toxicity to organs because of nanomaterials should be analyzed while administering intravenously [64]. In a study, it was reported that carboxyl group-functionalized multi-walled carbon nanotubes effectively enhanced the differentiation and mineralization of mammalian cementoblaststhrough interactions with the TGF-β/Smad pathway [65]. The activation of the intercellular effectors TGF-β/Smadand Akt/GSK-3βfollowed by the activation of the SNAIL-1 signaling pathway had an effect on the exposure to MWCNT-induced EMT, thus promoting pulmonary fibrosis [66]. Despite their role in various diseases, carbon nanotubes are used as biosensors for detecting various biomolecules. The separate deposition of graphene and multi-walled carbon nanotubes (MWCNT) on screen-printed carbon electrodes (SPE) offersthe electrochemical sensor core for the detection of bilirubin and is evaluated for electroanalytical parameters, such as higher sensitivity, wider linear range and lower detection limit. The data suggested that SPE-based bilirubin sensors can beessential for the management of healthcare and can be used for care diagnosis [67]. 

### 2.5. Metallic Nanoparticles

Metallic nanoparticles are 1–100 nm in size and include iron, gold, cobalt, nickel and oxides of cobalt ferrite, maghemite, magnetite, and the dioxide of chromium [5]. Gold nanoparticles (AuNPs) are excellent nanomaterials that are widely used in medicinal uses especially for cancer diagnosis and therapy. AuNP is composed of a surface withnegative reactive groups which functionwith the addition of therapeutic agents, ligands, etc., with a core gold atom. Metallic nanoparticles provide a platform for biomedical applications such as drug delivery, biomolecular sensing, and imaging [68]. Numerous molecules inclusive of biomolecules such as DNA, proteins, peptides and other therapeutic agents were used for functional chemical groups modifying the synthesis of metallic nanoparticles. In magnetic nanoparticles, due to their magnetic properties, a specific site in the body is targeted using an external magnetic field [5]. In this section, we have discussed some of the metallic nanoparticles and their significance in various disease conditions (Table 1).

A study was conducted to understand the role of metal nanoparticles (TiPs and CoPs) on Sirtuin 1 and showed that metal nanoparticles downregulated the expression of SIRT1 in macrophages which leads to osteolysis in THA failure. Metal nanoparticles downregulate SIRT1 in macrophages, whereas SIRT1 activation using resveratrol reduced the particle-induced expression of inflammatory cytokine nuclear factor kappa B (NF-κB) acetylation’sin vitro and in vivo osteolysis [79]. Genotoxicity was induced by metal nanoparticles (nano-Co and nano-TiO_2_) in A549 cells by inducing reactive oxygen species generation, stimulating DNA damage, and increasing the expression of phosphorylated histone H2AX (γ-H2AX), Rad51 and phosphorylated p53 [80]. Vascular-targeted AuNPs significantly improved the treatment efficacy of liposomal doxorubicin after radiation therapy by increasing the tumor delivery of chemotherapeutics and reducing the side effects of both RT and chemotherapy [81].Gold chrysophanol nanoparticles were reported to be effective in inhibiting the progression of human prostate cancer by reducing the activity of histone deacetylases and cell proliferation inhibition and cell cycle sub-G phase arrest-induced apoptosis. Additionally, a study reported that gold chrysophanol nanoparticles regulate cell cycle-related proteins, such as CDK1 and cyclin D1, via the activation of the AMPK and AKT pathways [82]. Greensynthesized AuNPs exerted their anticancer effect by inducing reactive oxygen species, stimulating mitochondrial dysfunction and enhancing caspase activation, ultimately leading to apoptosis, which suggests that this could be an effective cancer therapeutic strategy [83]. Phyto-engineered gold nanoparticles using theaqueous extract of *Acalypha indica* were tested for their antioxidant and wound healing potential in a BALB/c mice model with infected diabetic wounds. Nanoparticles showed increased antioxidant activity and re-epithelization was observed in the wound area which was fully covered by the nanoparticles, ultimately suggesting that gold nanoparticles can be used for the development of antibacterial and wound-healing agents [84]. Abdulrehman et al. [85] reported that silver–copper–boron (ACB) nanoparticles targetedagainst osteoblast infection, effectively decreased the toxicity of the system, and exhibited intracellular *S. aureus* activity when using ABC nanoparticles as bacterial agents. Silver nanoparticles 31.4 nm in size exerted cytotoxicity in the MCF-7 cancerous cells without damaging L-929 non-cancerous cells. Moreover, the synthesis of AgNPs showed an antagonist effect against standard and nosocomial strains of both Gram-negative and Gram-positive bacteria [86].

Green synthesized silver nanoparticles using the aqueous extract of *Fagonia indica* generate ROS and induce apoptosis via the activation of caspases 3 and 9 in MCF-7 cells [87]. The systemic administration of ironoxide+ peptide antisauvagine-30 decreased the withdrawal symptoms of amphetamine (including anxiety) without altering the movement of rats, suggestingASV-30’s anxiolytic activity was maintained with enhanced ASV-30 bioavailability. This suggests the effective neural distribution and increased efficacy of the nanoparticles [88]. Dextran-coated iron oxide nanoparticles with a cell-penetrating peptide are reported to be internalized into A549 and H358 cells and are activated by alternating magnetic fields.Data showed that the nanoparticles effectively stimulated the generation of reactive oxygen species, induced the depolarization of the mitochondrial membrane and the permeability of the lysosomal membrane, together with a high level of apoptosis by caspase 3/7 pathways in lung cancer cells [89]. In intracerebral hemorrhage rats, the intravenous injection of magnetosome-like ferrimagnetic iron oxide nanocube-6labelled with loaded human embryonic stem cell (ESC)-derived spherical neural masses (SNMs) effectively stimulatedthe brain upon the transportation of stem cells and decreased the severity of intracerebral hemorrhage (ICH) by recruiting neutrophils andmacrophages and downregulating pro-inflammatory cytokines, ultimately improving neurological function [90]. Copper oxide nanoparticles (CuONPs) induce cell death caused by oxidative DNA damage via stimulatingthe p38 MAPK inHUVECs, ultimately suggesting the release of copper ions induces vascular endothelial toxicity [91].

### 2.6. Quantum Dots

Quantum dotsare fluorescent, semiconductor nanocrystals (1–100 nm) consisting of an inorganic core structure comprising II–VI or III–V group elements of the periodic table and an aqueous organic shell such as ZnS [92]. A report suggested that there was an increasing energy gap with the decreasing size ofthe particles used; thus, smaller QDs emit light at higher energy and have a wide range of excitation spectra with narrow and tunable emission, and can thus be used as an optical signal in the detection of biomolecules including proteins, nucleic acids, bacteria, etc. [1]. Moreover, they possess high photostability, exert strong luminescence and act as superior agents when compared to other fluorescent materials. In this section, the crucial role of quantum dots in the field of biomedical application is discussed. The efficacy ofCdSe/ZnS core-shell-type quantum dot nanoparticles on cellular homeostasis was studied and the data showed that nanoparticles induced reactive oxygen species, altered antioxidant status and mitochondrial permeability together, along with increasing DNA fragmentation and enhancing apoptotic proteins, ultimately leading to blood–brain barrier damage [93]. In a study, an imaging probe was developed using a recombinant spike receptor-binding domain conjugated to fluorescent quantum dots (QDs) and the binding efficiency of spike proteins to the ACE2 receptor of infected cells was monitored. In the infected cells, the probe immediately binds to the cell’s surface and undergoes endocytosis while neutralizing antibodies completely prevent the binding and endocytosis process. Thus, quantum dots help in the screening and identification of inhibitors for coronavirus spike-mediated entry [94]. Despite their use, QDs act as a threatto human health, as reports showed that various organelles of human cells will be affected upon QD treatment, causing problems such as DNA damage, mitochondrial dysfunction, the rupture of lysosomes and endoplasmic stress. Apoptosis, autophagy and pyro-apoptosis signaling processes are activated by QD [95]. As cell tracking is necessary for understanding the mechanism of action of therapeutic products, carboxylated QD is reported to bind and be distributed within human bone marrow mesenchymal stem cells. QDs are efficiently localized inside mesenchymal stem cells as well as outside the cell in the extracellular matrix without additional staining methods and helps in tracking the migration of mesenchymal stem cells [96]. In a study, tumor-targeted liposomes were developed with QDs, as well as siRNA molecules together with anti-EGFR aptamers. This theranostic liposome helps in imaging the target cells or tumor xenograft mice by QDs and helps in the delivery of siRNA to the target cells [97]. In a study, CdTe/CdS quantum dots coated with lipids (QD-LC) were studied for their specificity on cancer cells. QD-LC stimulated apoptosis together with the activation of the JNK/caspase3 signaling pathway in HepG2 cells. Additionally, QD-LC inhibited tumor growth and delayed the formation of microtumors without causing damage to the other organs in mouse hepatocarcinoma H22 cells and thus, could act as an effective therapeutic agent for liver cancer [98]. Similarly, in another study, it was reported that gold–quantum dots inhibitthe growth of tumor-initiating cells by reducing metastatic events, blocking their self-renewal ability, inhibiting CTNNB1 signaling, and decreasing glioma spheroids, thus suggesting that gold–quantum dots help in eradicating residual resistant stem-like cells during cancer therapy [99]. Wei et al. [100] reported that PEG–graphene quantum dots, Pt, effectively sensitized oral squamous cell carcinoma by enhancing the accumulation of cisplatin (Pt) and stimulated the apoptosis and cell cycle arrest of cancer cells in both normoxia and hypoxia conditions. Additionally, nanoparticles carrying Pt inhibittumor growth with less systemic toxicity in the oral squamous cell carcinoma xenograft mouse cancer model. According to a study by Liang et al. [101], CdSe/ZnS QDs with polyethyleneimine (PEI) effectively transfected HepG2 cells with miR-26b plasmid andthe simultaneous upregulation of miR-26a expression, and inhibited cancer cellproliferation and cell cycle arrest stimulation, and the NPs ultimately exhibited both the ability for bioimaging and gene delivery. This suggests that QD NP could be engineered to enhance its efficiency as a gene therapy in the near future.

### 2.7. Nanospheres

Nanospheresaresolid polymeric nanoparticlesthat arepredominantly hydrophobic and which range in size from 100 to 300 nm. They are prepared using several methods including emulsification–evaporation, nanoprecipitation, etc., using biodegradable polymer materials including chitosan, gelatin and PLA. They are spherical matrices where the biological active compounds are distributed evenly and released to the environment with the help of the diffusion process [102]. They are important tools for drug delivery, with a high loading capacity for hydrophobic drugs, and they effectively protect the drugs from enzymatic and chemical degradation. Due to their hydrophobic surface, nanospheres are easily susceptible to opsonization and clearance by the reticulo-endothelial system (RES) and thus, surface modification is highly necessary to escape from RES [103]. In this section, we have discussed the importance of nanospheres in the field of medicine.

Inastudy, biodegradable poly(lactide-co-glycolide) withDC-cholesterol (PLGA/DC-Chol) nanospheres were prepared by encapsulating DNA and administered to rat spinal cords and mouse neural stem cells. The data showed an increased uptake of nanospheres with higher and long-lasting capacity. Furthermore, at the injured sites, angiogenesis was increased by the nanospheres, and the regeneration of tissue was also improved with recovering locomotor function; thus, PLGA/DC-Chol is suggested for reducingspinal cord injury usinggene deliveryvehicles [104]. Dual drug-releasing nanosphere-gels were prepared by entrapping both paeonol and salidroside and tested against ultraviolet B-induced melanogenesis. The doses of the drug were decreased to 50% when compared to individual compounds and it was reported that salidroside from the hydrogel inhibited melanocyte proliferation upon UVB radiation exposure, whereas paeonol inhibited tyrosinase activity. This ultimately suggests that the combined delivery of salidroside and paeonol might improve therapeutic efficacy against melanogenesis [105]. Selenium@SiO_2_ nanospheres were developed and tested against paraquat-simulatedacute lung injury. Nanospheres effectively decreased ROS, malondialdehyde, nuclear factor-κB, TNF-α and IL-1β, whilesuperoxide dismutase and glutathione levels were reduced. Ultimately, the authors suggested that Se@SiO_2_ nanospheres could be an effective therapeutic method against PQ poisoning [106]. Antibodies were immobilized in gelatin nanospheres by coupling between the antibodies and aminogroups of gelatin, andmolecular beacons (MB) were also incorporated into the nanospheres. Modified nanospheres were cultured with pro-inflammatory macrophages and it was observed that miRNA153-5p was effectively detected by MB-nanospheres by emitting fluorescence [107]. Ipriflavone-loaded mesoporous nanospheres are incorporated into endothelial progenitor cells. Nanospheres via clathrin-dependent endocytosis, phagocytosis and caveolae-mediated uptake enter into EPCs and induce the expression of VEGFR2 to stimulate and maintain angiogenesis [108]. Polypyrrole–Fe_3_O_4_ nanospheres are functionalized with herceptin and become internalized into cancer cells by endocytosis using HER-2, ultimately exerting anticancer activity by increasing cytotoxicity. Furthermore, in the same study, magnetization values of nanospheres were low in the fluid medium when compared to the solid state, suggesting that chemotherapeutic agents combined with magnetic carriers targeting cells could be an effective treatment for hyperthermia by killingcancer cellspreferentially [109].

The intratumoral injection of albumin nanospheres with zinc-phthalocyanine tetrasulfonate together with photodynamic therapy was tested against an Ehrlich solid tumor mouse model and it was reported that PDT with nanospheres induced tumor necrosis and inhibited tumor growth with minimal side effects and exerted anti-neoplastic activity. It was found that the effect was similar to that of intratumorallydoxorubicin-treated mice and thus, provides a nanosphere-based strategy for solid cancer therapy [110]. Poly(lactic-co-glycolic acid) nanospheres loaded with VEGF and glial cell line-derived neurotrophic factor (GDNF) effectively decreased the amount of amphetamine-induced rotation behavior andwas involved in neuronal recovery and protection in a partially lesioned Parkinson’s disease rat model [111]. In another study, the intraperitoneal administration of porous Se@SiO_2_ nanospheres effectively decreased ROS, inflammatory cytokines and proteins, and reduced tubular damage and apoptosis in ischemia/reperfusion (I/R)-induced acute kidney injury, thus representing a new treatment efficacy for kidney injury [112]. C-phycocyanin was loaded in nanospheres synthesized from carboxymethyl chitosan and CD55 and tested for the targeted delivery of C-phycocyanin in cervical cancer cells. Constructed nanospheres inhibited anti-apoptotic proteins and alternatively increased caspase3, decreased TGF-β, increased IL-6 and TNF-α and decreased tumor growth; nanospheres, thus, exerted an antitumor effect and acted as a potential tool for the development of the targeted treatment of cancer [113]. Chitosan-β-cyclodextrin-TPP-folicacid/alginate nanoparticles (spherically shaped) were loaded with curcumin and their effect on breast cancer cells was investigated. Curcumin nanoparticles effectively inhibited growth and enhanced the apoptosis of cancer cells. Thus, it was suggested that nanoparticles act as apotential tool for curcumin-targeted delivery in breast cancer cells [114]. Similarly in another study, gemcitabine-loadedalbumin nanospheres effectively decreased the proliferation and stimulated the apoptosis of human pancreatic cancer cells without causing any side effects when compared to the individual compound; thus, albumin nanospheres can acts as potential candidates to stimulate the anticancer effect of gemcitabine [115]. In a study, mesoporous silica decorated with gold nanoparticles exerted very high X-ray attenuation efficiency ~9.8 HU/mM in CT and also can be used as a effective vehicle for drug delivery [116]. In another study, mesoporous silica-coated gold nanorods loaded DOX induces toxicity in the breast cancer cells (MCF-7) and also decreased tumor growth in xenograft mice and ultimately suggested that nanoparticle could be an efficient drug delivering agent and also increases local heat at the tumor site and thus exhibited higher combined therapeutic potential [117].

## 3. Bioconjugation Process and Nanoparticles

**Figure 4:**
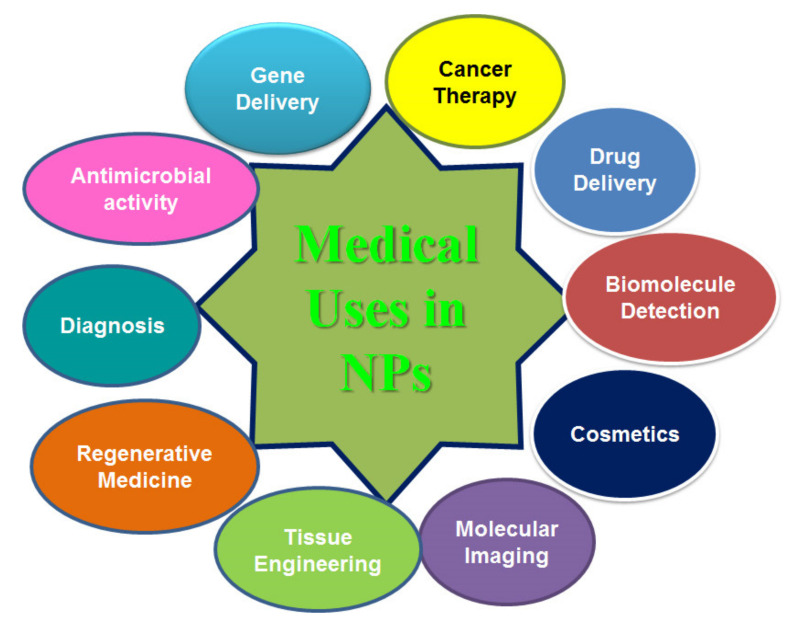
Broad spectrum biomedical application of nanoparticles.

Bioconjugation is the process of the covalent attachment of synthetic polymers to biological molecules such as nucleic acids, proteins, enzymes, carbohydrates, viruses or cells, and generates hybrid materials which help nanoparticles to interact specifically with biological systems. The bioconjugation of nanoparticles involves various methods such as covalent binding, nanoextraction/nanocondensation, adsorption, electrostatic interaction and non-covalent binding. Covalent binding is involved between thiol groups, primary amines, a carboxylic acid, maleimide, thiolhydrazide and aldehyde groups. The nanoextraction process occurs in the liquid phase where drugs should possess poor affinity towards solvents and a high affinity towards nanocarriers [118]. In the nanocondensation process, drug molecules migrate through the thin layer of a solvent and get adsorbed in the most active centers [118]. Drug agents can be accumulated on the external surface of nanocarriers via physical adsorption and electrostatic interactions [119]. Biological conjugation strategies use different attachment methods including (i) direct metal–sulfur or disulphide bonds, (ii) the crosslinking of functional groups, (iii) antibody linkers, (iv) streptavidin-biotin, and (v) DNA complementary base pairing [120]. Some important bioconjugated nanoparticles and their application in the diagnosis of various diseases are presented in Table 2. In a study, it was reported that the potassium ferricyanide-mediated oxidative coupling reaction is necessary for the attachment of aniline-functionalized AuNPs to oligonucleotides, peptides, and proteins [121]. Politi et al. [122] reported that gold nanoparticles are bioconjugated by a ligand exchange in which the surface of citrate-stabilizing gold NPs is exchanged with the thiolated peptide. Gold nanoparticles with horseradish peroxidase are conjugated via physical adsorption with the early six antibodies (CIP5) of human papilloma virus 16/18 to detect early 6 (E6) oncoproteins in cervical carcinoma progression [123]. In a study, polyethylene glycol-based ligands were covalently bound to the surface’s iron oxide particle via a siloxane group which improved the colloidal stability of the nanoparticles. Simultaneously, an antibody targeting plasminogen activator inhibitor-1 was coupled to the surface and its binding activity was assessed by ELISA [124]. In a study, radiobioconjugate preparation involved the doping of a radioactive agent and the attachment of the organic linker and therapeutic agent to the SPIONs’ surface which acted as efficient heat mediators and also exhibited a cytotoxic effect towards ovarian cancer cells (SKOV-3) expressing HER2 receptors [125]. An optimization study showed that *Listeria monocytogenes* polyclonal antibodies conjugate with gold nanoparticles by covalent binding and that the optimum condition necessary for bioconjugation is a pH of 6 and MES buffertype which enhance the stability of the antibody’s secondary structure [126]. Figure 4 shows the application of nanoparticles in medical fields.

Gold nanoparticles were noncovalently conjugated to a mouse monoclonal antibody specific to HER2 with an overexpression of SKBR3 breast carcinoma cellsby combining electrostatic and hydrophobic interactions of the antibody and the gold surface [136]. A novel transferrin-conjugated tumor-targeted, lipid–polymer hybrid nanoparticle formulation of plumbagin could suppress the growth of B16-F10 melanoma in vitro and in vivo [137]. Interleukin-10 (IL-10) is a key anti-inflammatory and immunosuppressive cytokine which acts as a therapeutic agent in inflammatory diseases; however, its therapeutic use has a short life span and increased proteolytic degradation. IL-10 bioconjugated covalently by carbodiimide crosslinker chemistry to PVP-coated silver nanoparticles (Ag-PVPs) and overcame itslimitations, thereby showing increased storage stability by preventing denaturation and improving its anti-inflammatory efficacy [138].

Surface functionalization of nanomaterials involves assembling of different organic and inorganic ligands in a nanoscale range by covalent bonds, hydrogen bonds, the electrostatic force etc. The most commonly used agents for surface functionlization are PEG, hyaluronic acid, polyvinyl alcohol, etc. [139]. Table 3 shows the surface functionalization of nanoparticle using various agents.

## 4. Nanoparticles Cytotoxicity

Nanomaterial can enter the cells through endocytosis, macropinocytosis, phagocytosis, diffusion, penetration. Generally, Nanoparticle with the size of 100 nm can enter the cells, 40 nm can enter nucleus and 35 nm can cross blood brain barrier. However, nanoparticle would exert toxicity by interacting with protein, lipids and nucleic acids and induces ROS, lipid peroxidation etc. Cationic nanoparticle can stimulate plasma membrane integrity, induces mitochondrial damage and lysosomal toxicity [147]. The physio-chemical properties of nanoparticle including size, shape, surface coating etc determines the toxicity of nanoparticle. Pacurari et al. [148] reported that single-wall carbon nanotubes stimulates oxidative stress and activates MAPK, NF-kB and AKT signaling pathway in normal mesothelial as well as malignant mesothelial cells in a dose dependent manner. By evaluating toxicological studies, it was reported that black phosphorus quantum dots at high concentration showed apoptotic effect on on HeLa cells while on normal cells nanoparticle induces lipid peroxidation, DNA breaks but it was recovered gradually [149]. In a zebra fish model, it was reported that silver nanoparticle induced cell death by inducing apoptosis, autophagy and modulated signaling pathways [150]. In spite of toxicity, nanoparticles are modified/engineered to decrease their toxicity on normal cells and increase toxicity specificially towards cancer cells.

## 5. Nanomaterials and Their Clinical Application

The use of nanomaterials in biomedicine has been exponentially raised over the last decade and many nanoparticles have entered clinical trial, some of which have been utilized for clinical application. With limited success in clinical trials, the abovediscussed nanoparticles have a significant role in medicine in terms of improving treatment strategies and diagnoses. In this section we will be discussing utilizing nanomaterials for bio-imaging, drug delivery, biosensing tissue engineering, medical devices, and immunoassays (Figure 1).

### 5.1. Biomedical Imaging

Medical imaging techniques are highly essential for early detection, diagnosis and therapeutic responses which would help in decreasing the mortality of patients and modifying therapeutic strategies (Table 4). Techniques that are currently in use include positron emission tomography (PET), magnetic resonance imaging (MRI), ultrasound (US), fluorescence-labeled imaging, computed tomography (CT), etc. [151]. In this section we have discussed the role of nanoparticles in imaging. CT is a commonly used imaging technique for screening cancer to distinguish between malignant and benign tumors; however, identifying the exact stage of cancer is considered an ongoing challenge. In a study, porous gold and solid gold nanoparticles were synthesized and administered to male rabbits to investigate the contrast in CT scans. The images revealed that porous gold nanoparticles effectively enhanced the contrast in both whole-rabbit and organ CT scans when compared to solid nanoparticles with higher efficacy [152]. In another in vivo study, gold nanoparticles were conjugated with anti-EGFR antibodies administered to both C57BL/6 mice with A431 subcutaneous tumors, and the nanoparticle accumulation in the mice was viewed using dual energy CT scans to measure the concentration of nanoparticles. CT scanning data showed that higher tumor gold accumulation was observed in the cetuximab-treated group when compared to traditional imaging, which suggested that gold nanoparticles act as better guides to improving the detection and diagnosis of lung cancer [135]. Superparamagnetic iron–platinum nanoparticles targeted with novel anti-Iba-1 together with MRI studied in a four-week post-stroke surviving rat brain were used to measure the spatio temporal changeson the microglial/macrophage. The data revealed that nanoparticle-enhanced MRI scans could be an effective monitoring method to understand the neuroinflammation development in live animals during the treatment of stroke as well as disease progression [153].

Ramaswamy et al. [163] showed that studying the cell distribution within tissue-engineered constructs through nondestructive methods is very difficult and it could be resolved by performing cell labeling using the superparamagnetic iron oxide (SPIO) contrast agent Feridex, which was approved by the FDA, together with transfecting agents to label chondrocytes and monitor them using MRI. Furthermore, the SPIOlabeling did not affect the phenotype or viability of the chondrocytes and could be used for tracking chondrocytes.In another study, anti-CD-133 antibodies added with ultrasmall superparamagnetic iron oxide were used to visualize cells along with MRI, and it was reported that HT29 xenograftMRIs showed hypotensive regions as clusters in CD133-expressing brain tumors; thus, nanoparticles together with MRI scans can be used for effective cell tracking and tumor detection [164]. In a rat model with sentinel lymph nodes (SLN), 68Ga-labelled magnetic nanoparticles were injected and monitored for the quantity of NP in each SLN rat. The data showed that PET detected all SLNs, whereas other SLNs were detected using magnetomotive ultrasound (MMUS) and MRI.Ultimately, the study showed that MMUS could be a standard pre-operative form of imaging with high spatial resolution [165]. PET imaging of a modified polyglucose nanoparticle using 18F-Macroflor was performed in macrophages to understand macrophage biology. A change in the population of macrophageswas detected using Macroflor PET imaging whereas molecular MRI showed changes in the inflammation.Ultimately, the data suggested thatMacroflor PET/MRI is a better clinical tool for studying macrophages accurately [166]. To understand the biodistribution and significance of the enhanced permeability and retention effect of nanoparticles in metastasis breast cancer patients, 19 patients in total diagnosed with metastatic HER2-positive breast cancer and administered 64Cu-MM-30 (64Cu-labeled HER2-targeted PEGylated liposomal doxorubicin) underwent two to three PET/CT scans, and theEPR effect and amount of nanoparticle deposition were quantified. The data suggested that the use of the pretreatment imaging of nanoparticle deposition helps patients to benefit from the therapeutic efficacy of nanoparticles [167]. Cerenkov radiation is used to image tissue; however, it is weak at detecting deeper tissue. Thus, a study has showed that the use of europium oxide nanoparticles effectively converted into fluorescence from the radioisotopes produced by Cerenkov and γ radiation and helps with detecting tumor lesions, reducing the uptake of radioactive tracersanddetecting small tumor lesions [168]. A study was designed to construct indocyanine green (ICG)-loaded nanobubbles by linking them with antigen peptide which was adhered to a prostate-specific membrane for the multimodal imaging of prostate cancer using ultrasound, and photoacoustic and fluorescence techniques. This nanoparticle effectively bound to PSMA-positive prostate cancer cells, LNCaP cellsand C4-2 cells, and also enhancedthe imaging potential of multimodal techniques. Ultimately, this multimodal contrast agent was found to helpwith prostate cancer diagnosis and assessment [169]. A study designed the USF contrast agents (ICG-NPs and ExiTron nano 12000) with particle sizes ranging from 70 nm to 330 nm and administered themintravenously to understand the distribution of contrast agents in tumor and normal tissues including liver, kidney, and spleen. Comparatively, ICG-NPs in the mice’s tumors and organsexhibited higher bio-stability, mostly in the liver and spleen, which were used for imaging in vivo [170].

### 5.2. Drug Targeting and Delivery

Nanoparticles or nanoformulations act as drug carriers in the targeting of drugs to the biological system. The targeting has been classified into active and passive processes. Active targeting involves the coupling of targeting molecule such as antibodies and peptides with a drug carrier inorder to anchor them to the receptor of target cells/tissue. On the other hand, in inactive targeting, the carrier drug enters the circulatory system and reaches the site of the target by attraction or binding. This is regulated by various factors, including pH, temperature, shape, molecular size, etc., coupling them to the structure of target site receptors. In this targeting, the complex drug carrier circulated in the bloodstream reaches the site of the target either by binding or affinity, and isinfluenced by factors such as shape, temperature, pH and molecular site. The important targeting molecules in the biological system include antigens, peptides, receptors and lipids of cell membranes, etc. However, the majority of nanoparticles/formulations are designed to target tumor tissue more so than other diseased organs/cells [171]. In this section, we will be discussingthe drug targeting and delivery of nanotechnology and its use in various diseases including cancer.

The nanoparticle, Angio-DOX-DGL-GNP, is intended to improve cancerous targeting by anchoring angiopep-2 to the nanoparticles’ surface, which would assistin accumulation and deep tumor penetrationwhen bounded to the protein that is related to the low-density lipoproteinreceptors that are overexpressed on triple-negative breast cancer cells. The effect of the penetration of deep tumors on active targeting leads to an increase in the inhibition rate of Angio-DOX-DGL-GNP on tumor growth in mouse models with 4T1 breast cancer tumors, suggesting that nanoparticles evolve nanoplatforms for breast cancer therapy [172]. Paclitaxel-loaded polylactide–polyethylene glycol (stealth) nanoparticles were tested for their anticancer activity. It was found that their cytotoxicity was enhanced in breast and ovarian cancer cell lines, and confocal microscopy imaging showed that nanoparticles are internalized into MCF-7 breast cancer cells within one hour, and in the xenograft model, nanoparticles were accumulated in the tumor tissue [173]. Celastrol-albumin nanoparticles were developed and tested for their anti-inflammatory effect in a nephritis model. The data revealed that nanoparticles effectively crossed the endotheliumand accumulatedin the mesangial cells, which resulted in decreased inflammation, extracellular matrix deposition, hypercellularity and proteinuria in a rat anti-Thy1.1 nephritis model. This ultimately suggests that it could be an effective treatment option for mesangioproliferative glomerulonephritis [174]. Lipoprotein-like nanoparticle-encapsulated lapatinib was designed and treated with BT-474 cells. The uptake of nanoparticles was effectively mediated through clathrin-dependent pinocytosis and macropinocytosis, and nanoparticles would diffuse into cytoplasm and induce cell death by causing cell cycle arrest and enhance apoptosis.

In xenograft mice bearing BT-474, the intravenous injection of nanoparticles was reported to target and get accumulated in the tumor site and ultimately induced permeability and retention in xenograft mice [175]. mPEG-PLGA-PLL nanoparticles (PEAL NPs) were constructed with ultrasound-targeted microbubble destruction and loaded with siRNA and tested for the delivery of siRNA into cells. The cellular uptake study showed that nanoparticlesare effectively internalized and can subsequently release siRNA into cells and thus, it was suggested that nanoparticles with ultrasound-targeted microbubble destruction are an effective strategy for siRNA-based therapeutics [176]. For the development of pulmonary drugs, albumin-based nanoparticles (NPs) loaded with albuterol sulfate were prepared and, upon evaluation of the efficiency of loading the drug, drug profiles and cell morphology, the researchers suggested that AS-BSA-NPs’ clinical development is a new drug delivery system and could prevent bronchospasm and improve the therapy in obstructive airway disease patients [177]. The overexpression of folic acid (FA)-linked polyamidoamine dendrimer (Den) nanoparticles loaded with *cis*-diamine platinum (CDDP) and HuR siRNA was evident through folate receptor-α (FRA) when distributed in H1299 lung cancer cells. The data showed that nanoparticles improved cytotoxicity in lung cancer cells without affecting normal MRC9 lung fibroblast cells and suggested that nanoparticles act as an effective carrier for the targeted delivery of siRNA or chemotherapy agents against cancer cells [178]. In another study, spermidine (Spd)-modified PLGA nanoparticles act as fluorofenidone carriers to understand the efficiency of anti-fibrosis in the lung. Nanoparticles increased high stability, increased loading and released the potential of nanoparticles to enhance the drug’s antifibrotic activity in the lungs. Fluorescence data showed that nanoparticles possess high affinity and get internalized into A549 cells via endocytosis and are distributed into the lung tissue. Histopathological data showed that nanoparticles effectively decreased the infiltration of neutrophils, alveolar collapse and reduced lung damage, and it was proposed that nanoparticles of Spd-AKF-PLGA might be effectively used for idiopathic pulmonary fibrosis treatment that acts as atargeted drug-delivery system [179].

### 5.3. Biosensors

The maintenance of the normal concentration of biomolecules including cholesterol, glucose, and proteins is necessary for the health of individuals, and exceeding or decreasing these values leads to serious health threats such as coronary disease, atherosclerosis, lipid dysfunction, diabetes, proteinuria, etc. [180]. Several analytical techniques are involved in identifying biomolecules such as spectroscopy, chromatography, and colorimetric methods. Still, these techniques have several disadvantages such as having poor specificity and selectivity, being time consuming and requiring a large number of samples, having a standardization problem, and the corrosiveness of the reagent, etc. [181]. Thus, cost-effective biosensor development with sensitivity, speed and selectivity is highly essential. Nanoparticles have attracted technological and scientific attention due to thesize of their effects, chemical stability and synthesis, leading to exclusive characteristics including catalytic, optical, and electronic efficiency that would increase analytical sensitivity, rapidity and selectivity [182]. Gold nanoparticle (AuNp)-coated nano-sized carbon interdigitated electrodes (IDEs) used to detect cholesterol by immobilizing cholesterol oxidase via diazonium cation electrochemical reduction. Developed biosensors for cholesterol with a 0.005–10 mM sensing range and high sensitivity suggest that cholesterol biosensors showed high specificity and selectivity for detecting cholesterol and could be used as diagnostic tools for detecting cholesterol-based diseases [183]. Silica nanoparticles (SNP) and indium oxide are self-assembled layer-by-layer on resistors with multi-site channel areas to develop glucose sensors. The SNP layer and glucose with immobilized glucose oxidase is detected within a chip at various sites, leading to concentration-dependent currents. The sensitivity is relative to resistor channel length, where a channel length of 5–20 μm is considered 4–12 nA/mM; by using sensor arrays, values will be obtained upon feeding sample solution, suggesting that nanoparticle biosensor arrays could be effective diagnostic tools for detecting glucose [184].

The integrated electrode with TiO_2_deposition on the surface along with activated (3-aminopropyl) triethoxysilane (APTES) was fixed with a single-stranded deoxyribonucleic acid (DNA) probe to develop biosensors to detect DNA. Upon the preparation of DNA sensors, *E. coli* O157:H7 was used for detecting target DNA and the binding degree between the target DNA and probe DNA, which was read using electrical signalsshown in a picoammeter. This assisted themeasurement of target DNA concentration. Due to probe DNA specificity, mismatched DNA, and complementary and non-complementary DNA can be distinguished and measured using this DNA sensor [185]. An amperometric H_2_O_2_ biosensor was developed on hemoglobin NPs’ covalent immobilization to a polycrystalline Au electrode (AuE), which could measure the amount of H_2_O_2_ in the serum of diabetic patients. The storage stability and rapid response was much higher in using HbNPs/AuEwhen compared to earlier sensors. Ultimately, the authors suggested that covalent-bound Np won AuE could be used for the development of other biosensors [186]. Prototype paper lateral flow biosensors were used to detect thefish nervous necrosis virus or whole particles (virions) of nodavirus. This biosensor was developedfor signal visualization using gold nanoparticles conjugated with polyclonal antibodies. After the homogenization and centrifugation of the brain and retina samples from fish, they can be directly applied on the sensor to detect the virus with increased efficiency and less non-specific interaction. This sensor can be used in the field of aquaculture and helps in disease monitoring and environmental safety [187].

Magnetic Fe_3_O_4_ nanoparticles (MNP) are incorporated into chitosan-functionalized graphene synthesized for differentusesother than biosensors such as MRI. Glucose oxidase is covalently linked with the carboxyl group of chitosan-functionalized graphene and the incorporation of MNP could facilitateenzyme loading. The developed glucose sensors possess a detection limit of 16 μM with 26 mM of glucose within a linear detection range. Thus, multifunctional MNP/CG nanoparticles can be used as biosensorsin vivo and in clinical areas such as MRI agents [188]. The electrochemical biosensor was developed using screen-printed electrodes and magnetic nanoparticles to detect ochratoxin A infeed samples and cereal with a detection limit of 0.01–0.82 ng/mL. It wassuggested that the sensor is less time consuming and has high sensitivity in ochratoxin A level determination [189]. To diagnose breast cancer, optical biosensors were developed for microRNA-155 (miR-155) and for that, a DNA probe was covalently bound to the negatively charged citrate-capped gold nanoparticle and target miRNA was adsorbed onto the positively charged polyethylenimine-coated gold nanoparticle which leads to hybridization and produces an optical signal. This sensor canidentify very low concentrations without the needfor expensive and time-consuming signal amplification methods [190]. DNA biosensors were developed using an aluminum interdigitated electrode coated with nanoparticles of crystalline titanium dioxide that measurethe electrical current response in the single-stranded *E. coli* O157:H7 DNA using a picoammeter. The (3-aminopropyl) triethoxysilane (APTES) functionalization to the sensorprovided a connection between carboxylate-probe DNA and the targeted DNA of *E*. *coli* O157:H7 that provides electrical signalsof 1.0 × 10^−13^ Mwithin the detection limit [191]. Immobilizing gold nanoparticles (AuNPs) on a surface using starch hydrogels resulted in a 400% increase in stability to the nanoparticles upon exposure tovarious environments, and areinvolved in interacting with macromolecules including DNA. For DNA sensors, the detection limit was reported to be 25 ng/mL and 75 ng/mL [192]. Surface plasmonic resonance (SPR) biosensors were developed by directly depositing label-free iron oxide nanoparticles (Fe_3_O_4_ NPs) together with paired antibodies to enhance the sensitivity and selectivity efficacy of biosensors in detecting α-synuclein in the serum of a sample of patients with Parkinson’s disease [193].Electrochemical glucose biosensorswere also developed with platinum nanoparticles (PtNPs) immobilized to the surface of glucose oxidase that was deposited on poly (Azure A). The sensor showed increased selectivity with a detection limit of 7.6 μMand high sensitivity towards glucose in patients’ samples, ultimately suggesting that it is a good alternative to glucose sensors when compared to existing sensors [194].

### 5.4. Tissue Engineering

Tissue engineering is an interdisciplinary field linking engineering, material science and biology that aims to design and develop biological tissue/organs in order to replace or retain biological samples. This can be achieved through a variety of biofabricationmethods including the use of hydrogels, 3D bioprinted scaffolds and nanotechnology. The major obstacles of tissue engineering include inappropriate biomaterial, improper cell growth, and the insufficient generation of growth factors. Nanoparticles, due to their distinct size-dependent properties, help in overcoming obstacles of tissue engineering [195]. Due to the nanoscale characteristics of biological systems, nanotechnology can be used to design bioassembled3D components and platforms and thus, can be used in life science and healthcare applications for producing desirable materials for successful cell delivery and tissue regeneration and minimizes immune responses and preventsinfection upon being implanted into the biological system [196].

The bone regeneration process requiresam engineered scaffold to mimic an extracellular matrix to provide an architecture to incorporate growth factors to induce cell recruitment and incorporate them into engineered scaffolds inorder to stimulate the osteogenic differentiation of stem cells. Bone morphogenetic protein 2’s (BMP-2)osteogenic growth factor is necessary for improving bone regeneration. Nanoparticles are necessary to enhance the proper delivery of BMP-2with no bioactivity loss and stay in the sitefor a longtimewhich would aid the formation of new bone. Poly(methyl methacrylate-co-methacrylic acid) nanoparticles incorporated with poly(ethylene glycol) dimethacrylate stimulate crosslinkers in new bone formation. Furthermore, BMP-2 assimilation into nanoparticles enhances the sustained release at the target site with unaffected bioactivity that is necessary for bone regeneration [197]. Poly(l-lactic acid) incorporated Silk fibroin (SF) nanoparticles could form scaffolds with the help of a phase-inversion technique that leads to a high affinity for albumin attachment and is thus, suggested for scaffolds in bone tissue engineering [198]. The membranes are made up of nanofibrous composition with glycolide, a copolymer of L-lactide, and diamond nanoparticles synthesized using an electrospinning technique. PLGA-ND membranes that are thicker andhave a smaller pore size showed higher mechanical resistance and helped in human osteoblast-like MG-63 cells attachment, migration, and proliferation which had a cell viability of 92 to 97% without any increase in the inflammatory protein. Thus, membranes of nanofibrous PLGA mixed withnanoparticles of diamond can be effectively used in bone tissue engineering [199]. In 2D cultures, layered double hydroxide (LDH) with MgAl-NO_3_ was developed into a vector that is nonviral which assisted the effective delivery of nucleic acids such as miRNA, siRNA and pDNA to mesenchymal stromal cells (MSCs). Additionally, LDH–miRNA nanoparticles incorporated into collagen-nanohydroxyapatite scaffolds which enhance the miRNA delivery andstimulate the overexpression in MSCs which act as excellent delivery platforms for tissue engineering application [200]. The scaffolds of polymers electrospun with a coating of nanoparticles of hydrophilic hematite and assembled layer-by-layer (LbL) wherea bioactive interface was generated between the cells and scaffolds, enhancing the improved hydrophilicity, increased stiffness and roughness and spread the cells on a nanoassembled scaffold. The seeded cells showed increased osteogenic marker genes which increased the alkaline phosphatase. The enhanced alkaline phosphatase (ALP) activities represented significantly enhanced osteogenic differentiation and thus, suggested that nanoassembled scaffolds are a new protocol in the engineering of bone tissue [201]. A biodegradable transparent scaffold was developed as a membrane of chitosan/polycaprolactone (PCL) with chitosan nanoparticles (CSNPs) for culturing corneal endothelial cells. After culturing, the transparency was similar to that of the stroma of human cornea and the cytotoxicity of the scaffold was reported as negative and increased the proliferation of endothelial cells. Thus, the developed scaffold can be effectively used for corneal endothelial regeneration [202]. Carboxylated branched poly(β-amino ester) nanoparticles stronglyaid in the delivery of cytosolic protein as well as gene editing using CRISPR-Cas9. Thus, the carboxylated branched poly(β-amino ester)s assemble themselves to form nanoparticles for the effective delivery of cytosolic protein. Nanoparticles enhance rapid cellular uptake and release functional cytosolic protein into cells. Additionally, nanoparticle-encapsulated CRISPR-Cas9 ribonucleoproteins would stimulate gene knockin and knockout in several cells, thus suggesting that this nanoparticle acts as a protein delivery tool, is involved in efficient gene editing and can be efficiently used for therapeutic applications [203]. Nanofibrous scaffolds were developed using poly(urethane), poly(ε-caprolactone) and gelatin, and were studied for immunological response. Nanofibrous scaffolds withstand the dynamic culture condition with increased cellular migration to the interior of a scaffold without increasing immunoreactivity; thus, it could be effectively used in tissue engineering applications [204]. A composite film was developed using poly(ε-caprolactone) and silicon-substituted hydroxyapatite nanoparticles, with the result that MC3T3-E1 were seeded andit was found that cell adhesion and growth was increased. In cell-seeded nanofilms, there was an increased generation of alkaline phosphatase and calcium content which is necessary for bone tissue engineering [205]. Similarly, mesoporous silica nanoparticles (MSNPs) and nanofibrous scaffolds PLGA/gelatin were embedded together and PC12 cells were cultured, favoringincreasedproliferation and cell attachment; thus, silica nanoparticlescaffolds could act as ideal supports for culturing stem cells and could be used for tissue engineering applications [206].

### 5.5. Immunoassays

To detect and quantify molecular biomarkers for disease diagnosis and treatment progression, various techniques are developed and among them, enzyme-linked immunosorbent assay (ELISA) uses immunocomplexes such as antibody–biomolecule enzymes which covert the reaction to product with an optical signal and the signal is related to the concentration or identification of the biomolecule in the biological sample. The limitations of various techniques such as ELISA are detection limit, sample volume, testing time, and sensitivity. Upon considering these limitations, nanotechnology is recently being developed to detect biomolecules with increased specificity and sensitivity using nanoparticle-based immunoassays. In this section, we will be discussing the immunoassays that usenanoparticles to detect various biomolecules. To detect mycobacterium tuberculosis MPT64 and CFP-10 proteins in the bodily fluids of TB patients, an MB-AuNP-I-PCR assay was designed using the functionalized gold nanoparticles (AuNPs) coupled with detection antibodies, oligonucleotides and magnetic beads (MBs), and found that sensitivity was much higher than magneto-ELISA and GeneXpert assays. This, thus, suggeststhat it leads to the development of a diagnostic kit [207]. In a study, a gold nanoparticle probe-based assay (GNPA) was developed for the detection of hepatitis C virus’ (HCV) core antigen. The monoclonal antibodies were coated on a magnetic microparticleprobe and then the NP-HCV core antigen-MMP sandwich immuno-complex was formed upon loading the target antigen protein and can be detected using a TaqMan probe-based real-time fluorescence PCR. This ultimately suggests that the improved GNPA decreased the interference of unbound barcode DNAs and could be a new method for HCV core antigen detection [208]. A comparative study was conducted on functionalized Au-NP-based iPCR (Nano-iPCR) with standard ELISA and iPCR to detect (IL)-3 and stem cell factor (SCF). The same immunoreagents (IL-3- and SCF-specific polyclonal antibodies and their biotinylated forms) were used throughout the assays. Nano-iPCR and iPCR are superior in sensitivity and detection range than ELISA. Specifically, Nano-iPCR assays detect rapidly using low concentrations of cytokines in complex biological fluids [209]. Coassembled nanoparticles (CPCI-NP) loaded with doxorubicin are reported to be effective in combination with photothermal/chemotherapeutic nanoplatforms against OSC-3 oral cancer. CPCI-NP loaded with imiquimod, an immunostimulant, is an effective treatment againstbreast cancer. Upon triple therapy, it not only kills light-irradiated primary tumors but also stimulates antitumor immunoactivity, suggesting that co-assembled nanoparticles could be used in photothermal and immuno-platforms for clinical translation [25]. In a study, it was reported that gold nanoparticle-coated starch magnetic beads were synthesized and functionalized using specific antibodies (immuno-AuNP@SMBs), which were found to be effective in separating and concentrating the target pathogenic bacteria, Escherichia coli O157:H7, and is also involved in surface-enhanced Raman scattering (SERS)-based detection. Thus, it is suggested that a SERS-based detection system helps in detecting pathogenic microorganism in clinical and food samples [210]. In another study, gold nanoparticles were functionalized with tau-specific monoclonal antibodies and an oligonucleotide template for an immuno-polymerase chain reaction (Nano-iPCR). Tau proteinsare quantified in the sample of cerebrospinal fluid and are compared with ELISA; ultimately, it was suggested that nano-iPCR is superior in the sensitivity and detection of tau proteins when compared to ELISA [211].

Dahiya et al. [212] designed gold nanoparticle-based RT-I-PCR for the detection of CFP-10 proteinof Mycobacterium tuberculosis in the clinical samples of TB patients. Comparatively, GNP-RT-I-PCR is relatively easy and more sensitive when compared with the streptavidin-biotin/succinimidyl-4-(N-maleimidomethyl) cyclohexane-1-carboxylate system employed in RT-I-PCR and thus, helps in the development of a TB diagnostic test kit. A gold nanoparticle (GNP) probe-based assay was developed for the detection of ricin, a potential biothreat agent. A chain of ricin was captured by a GNP probe coated with polyclonal antibodies and single-stranded signal DNA. An immuno-complex was formed by adding a magnetic microparticle (MMP) probe coated with ricin A chain monoclonal antibodies and was magnetically separated and characterized by PCR and real-time PCR. The data showed that the gold nanoparticle (GNP) probe-based assay was more sensitive than ELISA. Thus, it is suggested as a novel assay suitable for the ultrasensitive detection of proteins [213]. A nano-immuno test detects the *Mycobacterium avium* subspecies paratuberculosis (MAP) in the milk samples by using MAP-specific antibody-conjugated magnetic nanoparticles with resazurin dye as a chromogen. A ‘nano-immuno test’ in goat milk was 90.0% (sensitivity) and 92.6% (specificity) when compared to an IS900 PCR test [214]. An in-situ immuno-AuNP network-based ELISA biosensor was developed to detect pathogenic microorganisms with high sensitivity based on immuno-magnetic separation. This in-situ network biosensor was able to detect pathogens at low numbers such as 3 cells/mL of *Escherichia coli* O157:H7 and Salmonella typhimurium in buffer, whereas it was able to detect 3 CFU/mL and 15CFU/mL, respectively, in real samples [215]. A competitive immunoassay was developed using nonspecific monoclonal antibodies (mAb) against clenbuterol, whereas mAb is conjugated to fluorescent nanoparticles and free β-agonists compete for the binding sites. This helps in rapid screening with a detection limit of <50 pg g−^1^ [216]. Aly et al. [132] designed an immunoassay using gold nanoparticles grafted with PEG and functionalized with polyclonal antibodies raised against *C.* *sakazakii* to specifically target them. The specific binding of nanoparticles with cells was verified using transmission electron microscopy. It was shown that surface functionalization with anti-*C. sakazakii* optimizes the detection using extinction spectroscopy and reported the detection limit to be as low as 10 CFU/mL. Aflatoxin B1 was detected using competitive magnetic immunodetection (cMID) which is highly sensitive and portable. cMID was developed using immunofiltration columns coated with an aflatoxin B1-BSA conjugate with biotinylated aflatoxin B1-specific antibodies and magnetic particles that were functionalized with streptavidin. Ultimately, the assay showed a detection limit of 1.1 ng and suggested that it could be an effective method for decreasing the risk of processing contaminated commodities [217]. An electrochemical nanosensor was developed using fluorine-doped tin-oxide (FTO) for chlorpyrifos detection with AuNPs and anti-chlorpyrifos antibodies (chl-Ab). AuNPs providehigh electrical conductivity and specific resistivity and thus, increase the sensitivity of an immunoassay.In the FTO–AuNPs complex, chl-Ab was immobilized onto AuNPs’ surface to form a nanosensor thatexhibited high sensitivity and a detection limit for chlorpyrifos of up to 10fM [218]. 

### 5.6. Medical Devices

Nanomaterials have led to significant breakthroughs in the fields of bioengineering and medicine, and their need has been increased in the field of implantable medical devices and diagnostic devices (Table 3). In this section, we will be discussing the implants and diagnostic devices. VERIGENE^®^ System is the device used to detect nucleic acid and protein targets with rapid analysis and increased sensitivity. It uses a gold nanoparticle probe with a diameter of 13–20 nanometers (nm) that is functionalized with a defined number of DNA, RNA, and antibodies according to the assessment of target biomolecules (https://www.luminexcorp.com/verigene-nanogrid-technology/ (accessed on 15 January 2022).The Nanomix technology platform consists of carbon nanotubes and, upon binding to genes, proteins, or gas molecules, the electrical properties of the nanotubes are altered and detected. Nanomix Corporationdevelops sensors for detecting proteins, glucose, genetic variations, etc. Nanomix is currently involved in developing diagnostic devices for detecting SARS-CoV-2 antigens and antibody tests for COVID-19, stroke, and acute kidney disease (https://nano.com/nanomix.php (accessed on 15 January 2022). Altair Nanotechnologies developed Altair’s nanoscale titanium dioxide material-coated orthopedic implants [219], https://altairnano.com/products/ (accessed on 15 January 2022). Nanocomposite polymers including PLGA, polycaprolactone, and polyhedral oligomeric silsesquioxane poly-(carbonate-urea) are used to construct anti-thrombogenic and blood-compatible stents that would release drug release profiles along with a reduction in the platelet adhesion rate [220]. Another important diagnostic device is the pressure biosensor used for pressure-related diseases. It uses self-oriented nanocrystals in spatial arrangements to capture micropressure changes on cardiovascular walls [221]. Diamond-like carbon (DLC) surfaces containing silver nanoparticles are used for the modification of medical implants providing high stability and a high degree of biocompatibility. The immersion of implants in aqueous liquids results in rapid silver release that reduces the colonization or growth of surface-bound bacteria and subsequently facilitates the growth of silver-susceptible mammalian cells [222]. Nanostructures are composed of drug-filled liposomes and flexible magnetic nanochains. Due to their flexibility, magnetic nanochains undergo oscillation movements under the exposure to a radiofrequency field. This oscillation transfers the movements to the liposome, which is filled with doxorubicin, leading to the burst of liposomes and thesimultaneous release of the drug [223].

## 6. Conclusions

Nanotechnology helps society with early detection, effective drug delivery and the development of effective and low-cost home-based healthcare. It helps to interact with matter at the molecular scale, providing the possibility to understandbiomolecules and their pathways involved in diseases. Nanoparticles combined with therapeutic agents overcome problems that are associated with conventional therapy. Inspite of their biomedical application, some issues including side effects and toxicity are reported in many published data and are needed to be fully considered before their utilization in biological systems. It has been shown that nanoparticles suffer from problematic long-term stability, the leakage of drugs, non-specific distribution in the biological system and storage difficulties. Additionally, it is difficult to correlate the in vitro and in vivo analysis of nanoparticles, as in vitro and in vivo models are inadequate for the correlation. Another disadvantage of nanoparticles that needs to be considered is the inability of nanoparticles to crossbiological barriers such as the blood–brain barrier and intestinal barrier. It has been reported that more than 90% of the bioavailable dose of NPs fails to reach target organs due to sequestration by the liver or spleen. The physical handling of nanospheres is difficult in liquid anddry forms. Furthermore, due to the smaller size and larger surface area of nanoparticles, the chances of particle aggregation are increased. Therefore, extensive research needs to be conducted on the specific properties of therapeutic nanoparticles and their delivery strategies under both in vitro and in vivo conditions. We also suggest that improvements should be madeto the throughput cost of in vitro and in vivo assaysand flexibility, and many diagnostic nanodevices need to be investigated for the early diagnosis of diseases such as neurological disease, kidney injury, etc. Thus there is an urgent need for development of NP platform with modifiable/engineering characteristics like size, shape, charge that could be selectively used for various medicinal applications like drug delivery, diagnosis, therapeutic to improve the survival patients. Designing of more NPs platform with increased therapeutic efficacy for FDA approval and clinical use could improve patients outcome overall.

## Figures and Tables

**Table 1:** Phytochemical-coated nanoparticles in various diseases.

S. No.	Nanoparticles	Disease	In Vitro or In Vivo Studies	Mechanism	References
1.	NiO nanoparticles coated with phytomolecules using *Abutilon indicum* leaf extract.	Cervical cancer	HeLa cancer cells, Fibroblast cells.	Cytotoxicity andbiocompatibility; protects normal cells through their antioxidant potential.	[69]
2.	Gold nanoparticles using *Terminalia mantaly* extract	Colorectal cancer, breast and liver cancer	Caco-2, MCF-7 and HepG2, KMST-6 cells	Selective toxicity to cancer.	[70]
3.	Silver nanoparticles using *Alpinia calcarata* leaf extract	Bacterial infection	*E. coli*, *P. aeruginosa* (Gram negative) and *S. aureus* (Gram positive).	Antioxidant and antibacterial effect.	[71]
4.	Trimetallic Ag-Cu-Co nanoparticles using *Salvia officinalis* aqueous extract	Fungal infection	*Candida auris*	Induced apoptosis, stimulated cell cycle arrest (G2/M phase).	[72]
5.	Gold and silver nanoparticle using *Crassocephalum rubens*	Oxidative stress	In vitro determination	Scavenges free radicals and inhibits lipid peroxidation.	[73]
6.	Silver nanoparticles with *Boswellia dalzielii*	Leukemia	Kasumi-1 cells	Cell cycle arrest, antioxidant potential by scavenging free radicals.	[74]
7.	Se and Zn nanoparticles using *Ephedra aphylla* extract	Cancer	HePG-2 cells, HCT-116 cells, HeLa cells, WI-3 cell line	Cytotoxic effect. Antioxidant and antibacterial potential.	[75]
8.	Selenium dispersed in *P-Coumaric acid*	Rheumatoid arthritis	Wistar albino rats	Decreased lipid peroxidation, decreased TNF-α, IL-6, and MCP-1) upregulated MnSOD, Cu/ZnSOD, ECSOD, CAT, and GPx1.	[76]
9.	Ag–Au nanoparticles using *Asparagus racemosus* root extract	Inflammation	THP1 and NK92 cells	Decreased IL-1β, IL-6, and TNF-α IFN-γ.	[77]
10.	*Bacopa monnieri* phytochemicals, mediated synthesis of platinum nanoparticles	Parkinson’s disease	Zebrafish	Increased dopamine, reduced glutathione, glutathione peroxidase, catalase, SOD, reduced levels of malondialdehyde with enhanced locomotor activity.	[78]

**Table 2:** Nanoparticles in diagnostics.

S. No.	Nanoparticles	Purpose	In Vitro or In Vivo Studies	Mechanism	References
1.	Hsp70: superparamagnetic iron-oxide nanoparticle	Detection of experimental myocardium infarction	Male Wistar rats	Underwent sequential MRI scanning, accumulated in acture infarct.	[127]
2.	Reporter nanoparticle	Monitoring antitumor activity in real time	Breast cancer cells, ovarian cancer cells, and lung cancer cells. 4T1 breast cancer and Lewis lung carcinoma xenogratft Balb/c mouse model.	Nanoparticle entered into tumor via EPR effect. Upon release, drug stimulated apoptosis via caspase3 enzyme that cleaved the DEVD peptide and unquenched the fluorescent signal.	[128]
3.	DNA aptamer probe linked with azide–PEG nanoparticle	Detection of peptides and proteins	Plate-based assays	Signal brightness and stability higher compared to other labeling techniques, biocompatible, high affinity for targets, with less non-specific binding.	[129]
4.	Functionalized lanthanide oxide (Ln_2_O_3_) nanoparticles	MRI imaging	BALB/C-nude mice	Biocompatible, effective glioma-specific contrast agent	[130]
5.	PDDA and Au nanoparticle (AuNP)	Electrochemical immunosensor for interferon-gamma	Fabricated disposable ITO electrode and multiplexed electrochemical immunosensor	High sensitivity with a detection limit of 0.048 pg/mL	[16]
6.	Gold nanoshuttle	Detection	Immobilized into filter paper	Stronger extinction intensity at surface plasmon resonance peak, and exhibits much higher SERS activity. High SERS sensitivity in the detection of malachite green.	[131]
7.	Immunogold nanoparticle	Detection of *C. sakazakii*	* C. sakazakii *	Detection limit 10 CFU/mL.	[132]
8.	Folic acid-conjugated Gd_2_O_3_:Eu^3+^ nanoparticles	Detecting breast cancer	Breast cancer cells T-47D tumor xenograft	Less cell cytotoxic, get accumulated in the tumor tissue and used to detect breast cancer.	[133]
9.	Gold nanoparticle	Visualizing unlabeled gold nanoparticles	NU/NU (Crl:NU-Foxn1nu) nude mice	Identification and tracking of Au nanoparticles in vasculature (in real-time).	[134]
10.	Gold nanoparticle	Detecting lung cancer	A431 cells C57BL/6 mice and in nude mice (subcutaneous tumor)	Increased binding affinity with targeting ligands, increased tumor accumulation compared tothe circulation.	[135]

**Table 3:** Surface functionalization of nanoparticles.

S. No.	Nanomaterials	Surface Functionalization Agents	Purpose	References
1.	Cellulose nanocrystal	3-aminopropyltriethoxysilane	Exerts good thermal stability and a greater amount of residual char was formed at 500 °C.	[140]
2.	Negatively charged polystyrene nanoparticles	Sulfone or carboxyl groups,	Increased intestinal transport efficiency in caco2 cells.	[141]
3.	Hydrophobic nanoparticle	Amphiphilic polyaspartimide	Less than 12 nm hydrodynamic size, high colloidal stability, and biocompartibility.	[142]
4.	Starch nanocrystal	3-aminopropyl triethoxysilane	Uniform dispersion, improved hydrophobicity	[143]
5.	Poly(lactic-co-glycolic acid) nanoparticle	CarboxyPEG2000 and methoxyPEG550	Longevity in the blood and macrophage uptake.	[144]
6.	Gold nanoparticle	PEG	Biocompatability, biosafety	[145]
7.	Poly lactic-co-glycolic acid nanoparticle	peptide AC-1001 H3 (GQYGNLWFAY)	Enhance drug delivery	[146]

**Table 4:** Nanoparticles in molecular imaging/medical devices.

S. No.	Nanoparticles	Purpose	Studies	Mechanism	References
1.	Nanoparticle-coated microbubble	Ultrasound imaging	Releasing of nanoparticles using ultrasound-driven	The formation of stable nanoparticle-coated bubbles and controlled nanoparticle release using ultrasound.	[154]
2.	High-Z nanoparticles (ultrasmall bismuth nanoparticles)	Incorporated in medical devices (inferior vena cava filters (IVCFs))	Implanted adult domestic pig	Maintained increased contrast in high-energy single energy computer tomography for quantification. Coating the IVCF with bismuth NPs showed greater radiopacity than that of an uncoated IVCF. Able to differentiate between BiNP-IVCF and iodine contrast while injecting into the inferior vena cava of the pig.	[155]
3.	Selenium nanoparticle	Polymeric medical device coating	Se-coated substrate	Inhibited bacterial growth on PVC, PU and silicone and also reduced its function without using antibiotics.	[156]
4.	Nanostructured gold coating	Coating on medical devices	In situ studies	Antibiofouling potential Preventing the biofilm formation, clinical isolates and ATCC strains on medical devices	[157]
5.	Aqueous Stöber silica or iron oxide NPs	Fixing of polymermembrane to tissues	Wistar rats	Rapid closure and healing of deep wounds in skin and liver Inspite of blood flow, NPs can fix membranes to tissues.	[158]
6.	Np-incorporated perfluorocarbon microbubbles (MB)	Medical imaging	Fabrication of microfluidic devices	NP-incorporated MB can be detected using low-pressure ultrasound, and the monodispersed MB platform can be used for in vivo (10(6) MB/sec).	[159]
7.	Au-surface modified superparamagnetic core-shell NPs	Biosensor application	Biofunctional and spectroscopic characterization of superparamagnetic NPs	For bioseparation, NP can be directed using external magnetic fields	[160]
8.	FeO NP-Powered Micro-OCT	Imaging	In situ studies	Increased contrast, imaging and visualizing the real-time swelling process of polymeric MNs in biological samples using micro OCT Achievement of the best contrast-to-noise ratio using μOCT	[161]
9.	Varied-shaped gold nanoparticles	Coating for medical devices	Tested against *Candida albicans*, *Pseudomonas aeruginosa*, *Staphylococcus aureus* uropathogenic *Escherichia coli*	Increased bactericidal efficiency at nanogram doses, less toxicity can be coated on urological catheters	[162]

## Data Availability

Not applicable.
